# The chemical structure, pharmacological activity, and clinical progress of Gentianae Radix et Rhizoma

**DOI:** 10.3389/fphar.2025.1656493

**Published:** 2025-10-21

**Authors:** Hongfang Liu, Xiao Liu, Sheng Liu, Feng Zhao

**Affiliations:** ^1^ Pharmacy Department, Yantaishan Hospital, Yantai, Shandong, China; ^2^ School of Pharmacy, Yantai University, Yantai, Shandong, China

**Keywords:** gentianae radix et rhizoma, chemical structure, pharmacological activities, clinical applications, active constituents

## Abstract

As pivotal medicinal resources in the Gentiana genus (Gentianaceae), Gentianae Radix et Rhizoma exhibit remarkable chemical diversity and multi-target pharmacological activities. This review highlights *G. scabra* Bunge., *Gentiana rhodantha* Franch., *Gentiana manshurica* Kitag., *Gentiana veitchiorum* Hemsl., and other species, compiling 172 constituents from literature (2004–2024) and traditional sources: terpenoids (66 iridoids, 47 triterpenoids, and others), flavonoids, lignans, and alkaloids. Iridoids (e.g., gentiopicroside, swertiamarin) and triterpenoids are key bioactive agents. Pharmaco-logically, Gentiana extracts target NF-κB and MAPK pathways to suppress in-flammation and oxidative liver injury via Nrf2 activation, while inducing tumor cell apoptosis (Bax/Bcl-2) and S/G2-M phase arrest to inhibit lung/liver cancer proliferation. They enhance gastrointestinal repair, regulate motility, and mitigate chronic pain through central-peripheral analgesic synergy. Clinically, gentiopicroside demonstrates hepatoprotective, antiviral, and neuroprotective effects, with applications in herpes zoster, non-alcoholic fatty liver disease, and metabolic disorders. This review represents the first comprehensive integration of multidimensional chemi-cal-pharmacological-clinical data on Gentianae Radix et Rhizoma constituents, aiming to promote the expanded application of Gentianae Radix et Rhizoma in the pharma-ceutical field, provide a scientific basis for further development and utilization, and lay a foundation for subsequent research and industrial development.

## 1 Introduction

There are approximately 400 species of Gentiana, widely distributed in the temperate regions of the Northern Hemisphere and the alpine regions of the tropics, including Europe, Asia, the northern part of Australia and New Zealand, North America and along the Andes to Cape Horn, and the northern part of Africa. In China, there are about 247 species, found throughout the country, with most species concentrated in the mountainous areas of the southwest, primarily growing in alpine talus slopes, alpine meadows, and shrublands (Editorial Committee of Flora of China and Chinese Academy of Sciences, 1988; Wang et al., 2009; Che and Liang, 2008).

The Gentiana genus is composed of a diverse array of plants. Adhering to the principle of combining characteristics of vegetative and reproductive organs, the Gentiana genus is divided into 15 sections (Dong et al., 2017). The section Gentiana, represented by the traditional Chinese medicinal herb Gentianae Radix et Rhizoma, is known for its bitter taste and cold nature, which possess the effects of purging excess fire in the liver and gallbladder, as well as clearing damp-heat in the lower jiao. It is used in the treatment of diseases such as excessive heat in the liver meridian, convulsions, mania, jaundice, dysentery, Japanese encephalitis, sore throat, red eyes, scrotal swelling and pain, and damp itchiness in the genital area (Che and Liang, 2008; Jiao et al., 2024). Gentianae Radix et Rhizoma, derived from perennial herbs of the Gentianaceae family, was first recorded in the “Shen nong Bencaojing” during the Han Dynasty (202 B.C. – 220 C.D.) and is categorized as a moderate herb. It is known for its efficacy in treating cold and heat within the bones, convulsions, expelling noxious qi, healing severe injuries, stabilizing the functions of the five viscera, and detoxifying. Prolonged use is believed to enhance memory, invigorate the body, and delay aging (Liu, 2022). The 2020 edition of the “Chinese Pharmacopoeia” stipulates that Gentiana is the dried root and rhizome of *Gentiana manshurica* Kitag., *G. scabra* Bge., *Gentiana triflora* Pall., or *G. rigescens* Franch., which belong to the Gentianaceae family. These plants are perennial herbs, and their roots and rhizomes are used medicinally (Chinese Pharmacopoeia Commission, 2020).

As a traditional remedy for clearing heat and drying dampness, Gentianae Radix et Rhizoma demonstrates a variety of pharmacological actions in clinical settings. Modern pharmacological research indicates that Gentianae Radix et Rhizoma possesses hepatoprotective, choleretic, anti-inflammatory, analgesic, antimicrobial, antiviral, antiallergic, antitumor, neuroprotective, and stomachic activities. These diverse pharmacological effects are associated with its complex chemical structure, particularly with compounds such as gentiopicroside, swertiamarin, amarogentin, linarin, oleanolic acid, gentianine, and polysaccharides, highlighting the significant value of Gentianae Radix et Rhizoma (Jiao et al., 2024; Ning et al., 2017; Jiang et al., 2019; Xiao et al., 2019).

## 2 Chemical composition

For Gentianae Radix et Rhizoma are rich in diverse chemical constituents, primarily including terpenoids (iridoids and triterpenoids), flavonoids, lignans, and alkaloids. Among these, iridoids such as gentiopicroside, swertiamarin, and sweroside are not only the most abundant phytochemicals in Gentianae Radix et Rhizoma but also serve as key bioactive components responsible for their pharmacological activities (Song, 1986; Ye et al., 2023). Unless otherwise specified, all chemical constituents discussed in this section were isolated from roots/rhizomes, as defined in the Chinese Pharmacopoeia (2020). Compounds one to three were isolated from flowers of Gentiana rhodantha Franch. (non-pharmacopeial species), included for comparative chemical profiling.

### 2.1 Terpenoids

This article primarily describes 120 terpenoid compounds identified in *G. rigescens*, including 3 monoterpenes **(1–3)**, 68 iridoids **(4–71)**, 2 sesterterpenes **(72–73)**, and 47 triterpenoids **(74–120)**.

#### 2.1.1 Monoterpenoid

**FIGURE 1:**
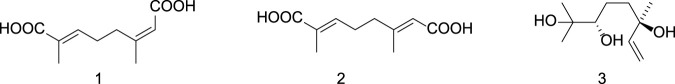
Chemical structures of Monoterpenoids in *Gentiana rhodantha* Franch.

Monoterpenoid compounds in Gentianae Radix et Rhizoma have been relatively less reported. Nevertheless, recent studies have gradually unveiled some monoterpenoid compounds within it. For instance, three monoterpenoid compounds were isolated from the flowers of *Gentiana rhodantha*, among which compound **1** is a novel compound, while compounds **2** and **3** are new natural products. These findings suggest that there may be more undiscovered monoterpenoid compounds in Gentianae Radix et Rhizoma, warranting further investigation. The ^1^H NMR and ^13^C NMR data for these compounds have been assigned (Zhou et al., 2023). Monoterpenoid compounds from Gentianae Radix et Rhizoma are presented in Table 1, and their chemical structures are illustrated in Figure 1.

**TABLE 1:** Monoterpenoids in *Gentiana rhodantha* Franch.

No.	Compound	Source	Ref.
1	(2E,6Z)-2,6-dimethyl-2,6-octadiene-1,8-dioic acid	*Gentiana rhodantha* Franch.	Zhou et al. (2023)
2	(2E,6E)-2,6-dimethyl-2,6-octa-diene-1,8-dioic acid	*Gentiana rhodantha* Franch.	Zhou et al. (2023)
3	(3S,6R)-dimethyloct-7-ene-2,3,6-triol	*Gentiana rhodantha* Franch.	Zhou et al. (2023)

#### 2.1.2 Iridoids

**FIGURE 2:**
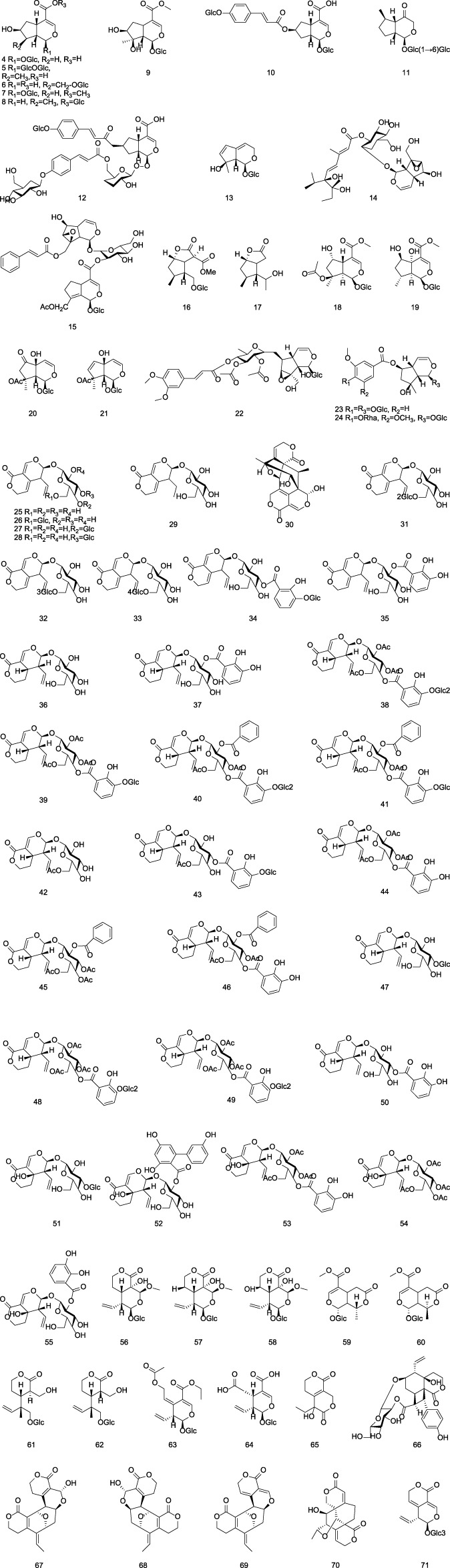
Chemical structures of iridoids in Gentianae Radix et Rhizoma.

The majority of iridoid components in Gentianae Radix et Rhizoma are secoiridoids, which are commonly glycosylated at the C-1 position. The isolated iridoids are primarily classified into regular iridoids, 4-demethylated iridoids, and secoiridoids (Ye et al., 2023). The regular iridoids include loganic acid **(4)**, 6′-O-β-D-glucopyranosyl loganic acid **(5)**, and loganin **(7)**; the 4-demethylated iridoids include Scrophulariadioside A **(22)**, Rehmannioside B **(23)**, Rehmannioside C **(24)**; and the secoiridoids include gentiopicroside **(25)**, Gentiotrifloroside **(36)**, and Swertiamarin **(51)**. For detailed information on the iridoid components in Gentianae Radix et Rhizoma, refer to Table 2 and Figure 2.

**TABLE 2:** Iridoids in Gentianae Radix et Rhizoma.

No.	Compounds	Sources	Ref.
4	Loganic acid	*Gentiana pedicellata*	Liu (2004)
5	6'-O-β-D-glucopyranosyl loganic acid	*Gentiana rhodantha* Franch.	Xu et al., 2007
6	Gentiopicroside C	*Gentiana manshurica* Kitag.	Zhou et al. (2017)
7	Loganin	*Gentiana scabra* Bunge.	Xu et al. (2007)
8	Loganic acid 11-O-β-D-glucopyranosyl ester	*Gentiana scabra* Bunge.	Xu et al. (2007)
9	Caryoptoside	*Gentiana scabra* Bunge.	Xu et al. (2007)
10	4''-O-β-D-glucopyranosyllinearoside	*Gentiana scabra* Bunge.	Wang et al. (2017)
11	Gentianaside	*Gentiana scabra* Bunge.	Xu et al. (2007)
12	4''-O-β-D-glucosyl-6'-O-(4-O-β-D-glucosyl- caffeoyl)linearoside	*Gentiana manshurica* Kitag.	Yu (2006)
13	Tianmu Dihuang glycoside A	*Gentiana manshurica* Kitag.	Zhou et al. (2017)
14	Tianmu Dihuang glycoside E	*Gentiana manshurica* Kitag.	Zhou et al. (2017)
15	Globuloside A	*Gentiana triflora* Pall.	Olennikov et al. (2015)
16	Cornusoside A	*Gentiana triflora* Pall.	Olennikov et al. (2015)
17	Cornolactone A	*Gentiana triflora* Pall.	Olennikov et al. (2015)
18	6,9-epi-8-O-acetylshanziside methyl ester	*Gentiana triflora* Pall.	Olennikov et al. (2015)
19	5,9-epi-Penstemoside	*Gentiana triflora* Pall.	Olennikov et al. (2015)
20	6-keto-8-acetylhookgrass glycoside	*Gentiana scabra* Bunge.	Olennikov et al. (2015)
21	6,7-dehydro-8-acetylhookgrass glycoside	*Gentiana scabra* Bunge.	Olennikov et al. (2015)
22	Scrophulariadioside A	*Gentiana manshurica* Kitag.	Zhou et al. (2017)
23	Rehmannioside B	*Gentiana manshurica* Kitag.	Zhou et al. (2017)
24	Rehmannioside C	*Gentiana manshurica* Kitag.	Zhou et al. (2017)
25	Gentiopicroside	*Gentiana scabra* Bunge.	Zhang et al. (2019)
26	2H-gentiopicroside	*Gentiana rigescens* Franch.	Ye et al. (2018)
27	6'-O-β-D-glucopyranosylgentiopicroside	*Gentiana scabra* Bunge.	Zhang et al. (2019)
28	4'-O-β-D-glucopyranosylgentiopicroside	*Gentiana scabra* Bunge.	Zhang et al. (2019)
29	Olivieroside C	*Gentiana crassicaulis*	Sun and Xia (1984)
30	(−)-Swertiamarigenin a	*Gentiana scabra* Bunge.	Wang (2014)
31	Scabrans G3	*Gentiana scabra* Bunge.	Zhang et al. (2019)
32	Scabrans G4	*Gentiana scabra* Bunge.	Zhang et al. (2019)
33	Scabrans G5	*Gentiana scabra* Bunge.	Zhang et al. (2019)
34	Gentiotrifloroside	*Gentiana scabra* Bunge.	Jiang et al. (2008)
35	2'-(2,3-dihydroxybenzoyl) Gentiopicroside	*Gentiana rigescens* Franch.	Zhao et al. (2009)
36	Gentiotrifloroside	*Gentiana scabra* Bunge.	Guo and Piao (2011)
37	2'-(o,m-dihydroxybenzyl)sweroside	*Gentiana scabra* Bunge.	Guo and Piao (2011)
38	4'''-O-β-D-glucosyltrifloroside	*Gentiana scabra* Bunge.	Wang (2015)
39	Trifloroside	*Gentiana scabra* Bunge.	Hu and Li (2002)
40	Rindoside	*Gentiana scabra* Bunge.	Li et al., 2011
41	4'''-O-β-D-glucosylscabraside	*Gentiana scabra* Bunge.	Wan (2022)
42	6'-O-acetylsweroside	*Gentiana straminea*	Zhu et al. (2021)
43	6'-O-acetyl-3'-O-[3-(β-D-glucopyranosyloxy)-2-hydroxybenzoyl]sweroside	*Gentiana manshurica* Kitag.	Yu (2006)
44	(1S,5R,9R)-deglucosyltrifloroside	*Gentiana triflora* Pall.	Li and Wang (2023)
45	(1S,5R,9R)-scabraside	*Gentiana triflora* Pall.	Li and Wang (2023)
46	Deglucoscabraside	*Gentiana triflora* Pall.	Li and Wang (2023)
47	3'-O-β-D-glucopyranosyl sweroside	*Gentiana scabra* Bunge.	Zhang (2015)
48	6'-O-β-D-glucopyranosyl loganic acid	*Gentiana rigescens* Franch*.*	Liu et al. (2024)
49	Scabraside	*Gentiana rigescens* Franch.	Liu et al. (2024)
50	3'-(2,3-dihydroxybenzoyl) gentian glycoside	*Gentiana rigescens* Franch.	Zhao et al. (2009)
51	Swertiamarin	*Gentiana scabra* Bunge.	Hu and Li (2002)
52	Amaroswerin	*Gentiana scabra* Bunge.	Zhang, 2015
53	Deglucogelidoside	*Gentiana scabra* Bunge.	Li and Wang (2023)
54	Swertiamarin tetraacetate	*Gentiana scabra var. Buergeri*	Li et al. (2024)
55	Gentiascabraside A	*Gentiana scabra* Bunge.	Guo and Piao (2011)
56	6β-hydroxyswertiajaposide A	*Gentiana scabra* Bunge.	Zhang et al. (2019)
57	2'-(2,3-dihydroxybenzoyl) Gentiopicroside	*Gentiana scabra* Bunge.	Zhang et al. (2019)
58	Swertiajaposide A	*Gentiana scabra* Bunge.	Zhang et al. (2019)
59	Honeysuckle glycoside	*Gentiana scabra* Bunge.	Xu et al. (2007)
60	8-epi-kingiside	*Gentiana scabra* Bunge.	Xu et al. (2007)
61	1-O-β-D-glucosyl-4-epiamplexine	*Gentiana scabra* Bunge.	Zhang et al. (2019)
62	1-O-β-D-glucopyranosylamplexine	*Gentiana scabra* Bunge.	Zhang et al. (2019)
63	Gentiorigenoside A	*Gentiana scabra* Bunge.	Liu et al. (2024)
64	Secologanoside	*Gentiana rhodantha* Franch.	Xu et al. (2007)
65	Gentiolactone	*Gentiana scabra* Bunge.	Li and Wang (2023)
66	Rigenolide A	*Gentiana rigescens* Franch.	Zhao et al. (2009)
67	(+)-Gentiovarisin a	*Gentiana scabra* Bunge.	Zhang et al. (2023)
68	(-)-Gentiovarisin a	*Gentiana scabra* Bunge.	Zhang et al. (2023)
69	Gentiovarisin B	*Gentiana scabra* Bunge.	Zhang et al. (2023)
70	Villoside	*Gentiana scabra* Bunge.	Wang (2014)
71	Villosolside *B*	*Gentiana scabra* Bunge.	Wang (2014)

#### 2.1.3 Sesterterpenoids

**FIGURE 3:**
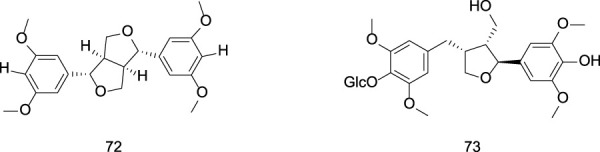
Chemical structures of sesterpenoids in *Gentiana scabra* Bunge.

Sesterterpenoid compounds found in gentians are similar in scarcity to monoterpenoids. Liu et al. identified the sesterterpenoid pranferin from a methanol extract of *G. scabra* Bunge. (Liu, 2004). Specific information on sesterterpenoid constituents from Gentiana can be found in Table 3 and Figure 3.

**TABLE 3:** Sesterterpenoid in *Gentiana scabra* Bunge.

No.	Compounds	Sources	Ref.
72	(+)-Syringaresinol	*Gentiana scabra* Bunge.	Ye et al. (2023)
73	Pranferin	*Gentiana scabra* Bunge.	Li (2023)

#### 2.1.4 Triterpenoids

**FIGURE 4:**
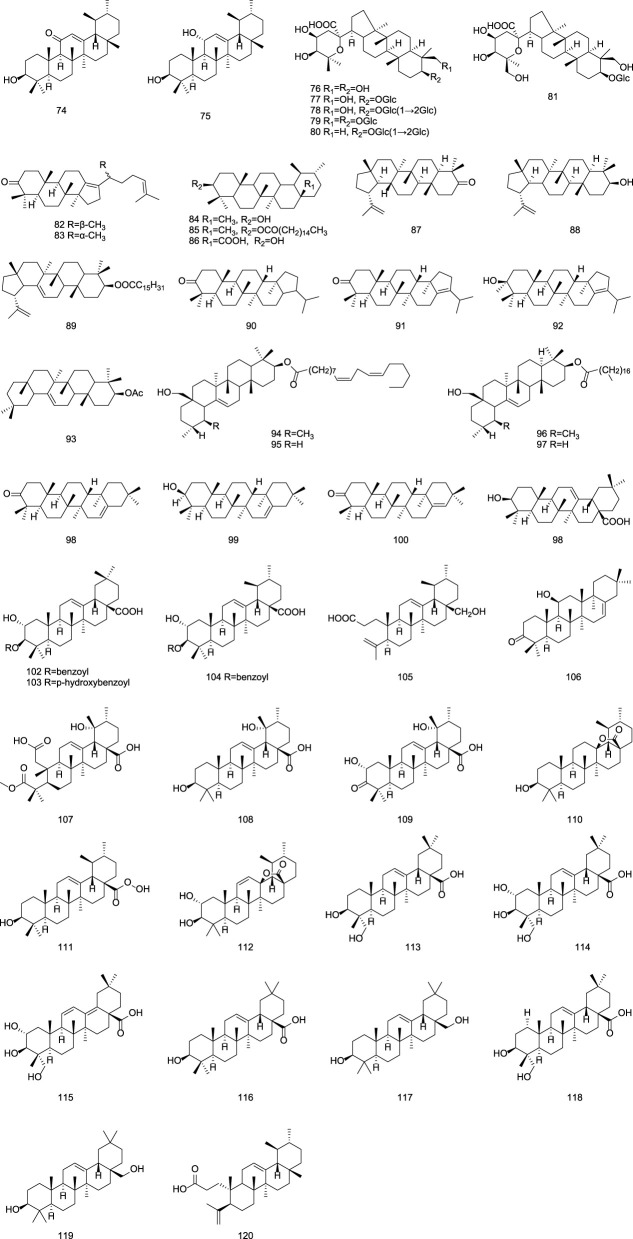
Chemical structures of triterpenoids in Gentianae Radix et Rhizoma.

Triterpenoids isolated from Gentiana plants mainly include dammarane-type compounds, characterized by a β-configured angular methyl group at the C-8 position, such as gentirigenic acid (**76**), gentirigeoside A (**77**), and gentirigeoside B (**78**); ursane-type compounds, with A/B, B/C, and C/D rings in a trans configuration and D/E rings mostly in a cis configuration, such as α-amyrin (**84**), α-amyrin palmitate (**85**), and ursolic acid (**86**); and lupane-type compounds, featuring a five-membered E ring with isopropyl substitution, such as 17β,21β-epoxyhopan-3-one (**90**), hop-17 (21)-en-3-one (**91**), and hop-17 (21)-en-3β-ol (**92**). Specific information on triterpenoid constituents from Gentianae Radix et Rhizoma can be found in Table 4 and Figure 4. Additionally, purified dammarane-type triterpenoids from Gentiana rigescens exhibited *in vitro* antifungal activity against Glomerella cingulata (inhibition zones: 0.8–2.0 cm) in a disk diffusion assay at 1 mg/mL, with Carbendazim as a positive control after 72 h of incubation (Xu et al., 2007).

**TABLE 4:** Triterpenoids in Gentianae Radix et Rhizoma.

No.	Compounds	Sources	Ref.
74	3β,11α-dihydroxyurs-12-ene	*Gentiana scabra* Bunge.	Wang et al. (2023)
75	3β-hydroxy-11-oxours-12-ene	*Gentiana scabra* Bunge.	Wang et al. (2023)
76	Gentirigenic acid	*Gentiana rigescens* Franch.	Yang and Wang (2005)
77	Gentirigeoside A	*Gentiana rigescens* Franch.	Yang and Wang (2005)
78	Gentirigeoside B	*Gentiana rigescens* Franch.	Yang and Wang (2005)
79	Gentirigeoside C	*Gentiana rigescens* Franch.	Yang and Wang (2005)
80	Gentirigeoside D	*Gentiana rigescens* Franch.	Yang and Wang (2005)
81	Gentirigeoside E	*Gentiana rigescens* Franch.	Yang and Wang (2005)
82	(20S)-dammara-13(17),24-dien-3-one	*Gentiana scabra* Bunge.	Cao et al. (2012)
83	(20R)-dammara-13(17),24-dien-3-one	*Gentiana scabra* Bunge.	Cao et al. (2012)
84	Α-amyrin	*Gentiana rhodantha* Franch.	Xie et al. (2021)
85	Α-amyrin palmitate	*Gentiana rhodantha* Franch.	Xie et al. (2021)
86	Ursolic acid	*Gentiana scabra* Bunge.	Yu (2006)
87	Lupeolone	*Gentiana manshurica* Kitag.	Yu (2006)
88	Lupeol	*Gentiana manshurica* Kitag.	Yu (2006)
89	Lupeol palmitate	*Gentiana scabra* Bunge.	Cao et al. (2012)
90	17β,21β-epoxyhopan-3-one	*Gentiana scabra* Bunge.	Cao et al. (2012)
91	Hop-17(21)-en-3-one	*Gentiana scabra* Bunge.	Cao et al. (2012)
92	Hop-17(21)-en-3β-ol	*Gentiana scabra* Bunge.	Cao et al. (2012)
93	Β-amyrin acetate	*Gentiana scabra* Bunge.	Chen et al. (2008)
94	Uvaol 3-O-linoleate	*Gentiana scabra* Bunge.	Jia et al. (2012)
95	Erythrodiol 3-O-linoleate	*Gentiana scabra* Bunge.	Jia et al. (2012)
96	Uvaol 3-O-stearate	*Gentiana scabra* Bunge.	Jia et al. (2012)
97	Erythrodiol 3-O-stearate	*Gentiana scabra* Bunge.	Jia et al. (2012)
98	Chirat-16-en-3-one	*Gentiana scabra* Bunge.	Cao et al. (2012)
99	Chiratenol	*Gentiana manshurica* Kitag.	Yu (2006)
100	Chirat-17(22)-en-3-one	*Gentiana scabra* Bunge.	Cao et al. (2012)
101	Oleanolic acid	*Gentiana scabra* Bunge.	Li (2023)
102	3β-O-benzoyl-2α-hydroxyolean-12-en-28-oic acid	*Gentiana scabra* Bunge.	Kikuchi et al. (2005)
103	3β-O-(4′-hydroxybenzoyl)-2α-hydroxyolean-12-en-28-oic acid	*Gentiana scabra* Bunge.	Kikuchi et al. (2005)
104	3β-O-benzoyl-2α-hydroxyurs-12-en-28-oic acid	*Gentiana scabra* Bunge.	Kikuchi et al. (2005)
105	3,4-seco-ursan-28-hydroxy-12-en-3-oic acid	*Gentiana scabra* Bunge.	Kikuchi et al. (2005)
106	11β-hydroxy-chairat-16-16-en-3-one	*Gentiana scabra* Bunge.	Kikuchi et al. (2005)
107	19-hydroxy-2,3-seco-urs-12-ene-2,3,28-trioic acid 3-methyl ester	*Gentiana rhodantha* Franch.	Zhou et al. (2023)
108	Pomolic acid	*Gentiana rhodantha* Franch.	Zhou et al. (2023)
109	2α,19α-dihydroxy-3-oxo-urs-12-ene-28-oic acid	*Gentiana rhodantha* Franch.	Zhou et al. (2023)
110	Ursolic acid lactone	*Gentiana rhodantha* Franch.	Zhou et al. (2023)
111	Uvaol	*Gentiana rhodantha* Franch.	Zhou et al. (2023)
112	Ilelatifol D	*Gentiana rhodantha* Franch.	Zhou et al. (2023)
113	Hederagenin	*Gentiana rhodantha* Franch.	Zhou et al. (2023)
114	2α,3β,23-trihydroxy-12-ene-28-oleanolic acid	*Gentiana rhodantha* Franch.	Zhou et al. (2023)
115	2α,3β,23-trihydroxyoleana-11,13(18)-dien-28-oic acid	*Gentiana rhodantha* Franch.	Zhou et al. (2023)
116	Oleanolic acid	*Gentiana rhodantha* Franch.	Zhou et al. (2023)
117	Ursolic aldehyde	*Gentiana rhodantha* Franch.	Zhou et al. (2023)
118	2α,3β,24-trihydroxy-12-ene-28-oleanolic acid	*Gentiana rhodantha* Franch.	Zhou et al. (2023)
119	Erythrodiol	*Gentiana scabra* Bunge.	Wang et al. (2023)
120	Roburie acid	*Gentiana scabra* Bunge.	Wang et al. (2023)

### 2.2 Flavonoid

**FIGURE 5:**
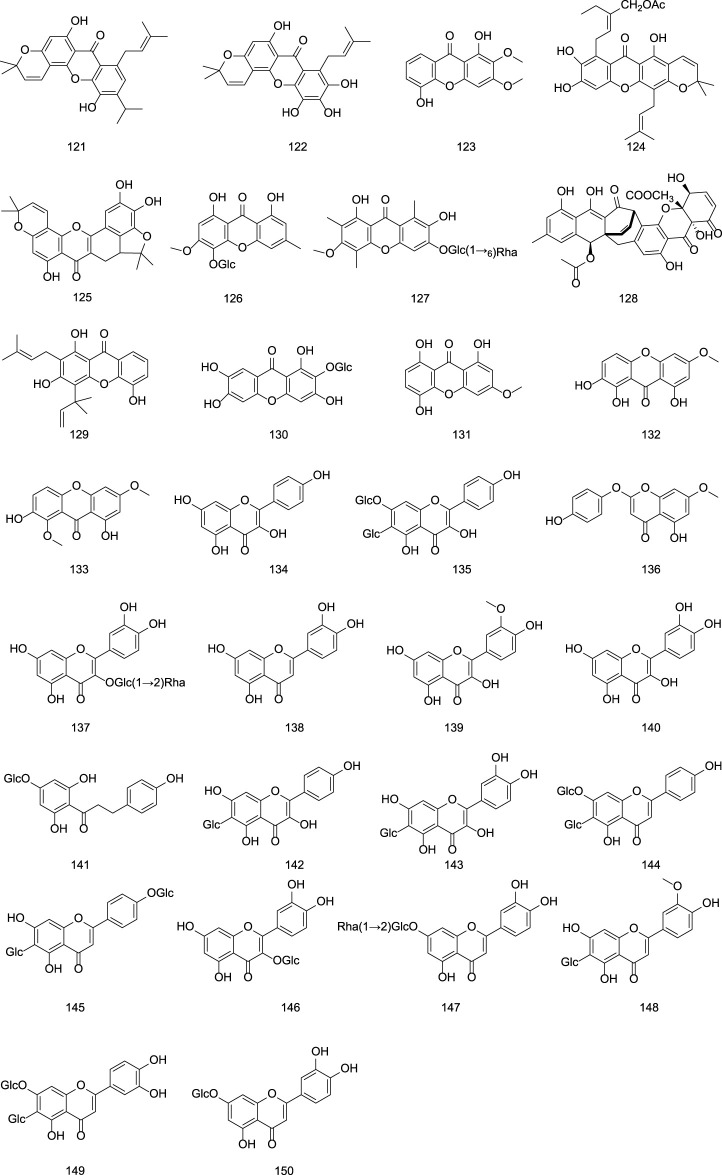
Chemical structures of flavonoids in Gentianae Radix et Rhizoma.

The flavonoid constituents in *G. rigescens* are primarily composed of benzochromones and flavonoid glycosides. Representative compounds include oliganthaxthanone A **(121)**, pinetoxanthone **(122)**, mangiferin **(130)**, luteolin **(138)**, kaempferol **(134)**, quercetin **(140)**, isoorientin **(142)**, isovitexin **(143)**, and their derivatives (Liu, 2004; Zhou et al., 2017; Wang, 2015; Yu, 2006; Olennikov et al., 2015). as shown in Table 5 and Figure 5.

**TABLE 5:** Flavonoid in Gentianae Radix et Rhizoma.

No.	Compounds	Sources	Ref.
121	Oliganthaxthanone A	Gentiana manshurica Kitag.	Wu et al. (2017)
122	Oliganthaxthanone B	Gentiana manshurica Kitag.	Wu et al. (2017)
123	1,5-dihydroxy-2,3,4-trimethoxyxanthone	Gentiana manshurica Kitag.	Wu et al. (2017)
124	Bannaxanthone I	Gentiana manshurica Kitag.	Wu et al. (2017)
125	Artomandin	Gentiana manshurica Kitag.	Wu et al. (2017)
126	Polyhongkongenosides A	Gentiana manshurica Kitag.	Wu et al. (2017)
127	Acremoxanthone D	Gentiana manshurica Kitag.	Wu et al. (2017)
128	Sporormielloside	Gentiana manshurica Kitag.	Wu et al. (2017)
129	Pinetoxanthone	Gentiana manshurica Kitag.	Wu et al. (2017)
130	Mangiferin	Gentiana manshurica Kitag.	Li X. et al. (2018)
131	Arnicaefolin	Gentiana straminea	Li X. et al. (2018)
132	Gentiakochianin	Gentiana straminea	Li X. et al. (2018)
133	Gentian xanthone phenol	Gentiana straminea	Li X. et al. (2018)
134	Kaempferol	Gentiana crassicaulis	Li (2023)
135	Saponarin	Gentiana scabra Bunge.	Lian et al. (2018)
136	6-Demethoxy-7-methylcapillarisin	Gentiana scabra Bunge.	Lian et al. (2018)
137	Rutin	Gentiana scabra Bunge.	Zhang et al. (2014)
138	Luteolin	Gentiana olivieri	Zhang et al. (2014)
139	Isorhamnetin	Gentiana olivieri	Zhang et al. (2022)
140	Quercetin	Gentiana scabra Bunge.	Li et al. (2015)
141	Trifolirhizin	Gentiana scabra Bunge.	Isakovic et al. (2008)
142	Isovitexin	Gentiana scabra Bunge.	Li X. et al. (2018)
143	Isoorientin	Gentiana scabra Bunge.	Li X. et al. (2018)
144	Isovitexin-7-O-glucoside	Gentiana scabra Bunge.	Li X. et al. (2018)
145	Isosakuranetin	Gentiana scabra Bunge.	Ruan et al. (2015)
146	Hyperin	Gentiana scabra Bunge.	Yang et al. (2018)
147	Lonicerin	Gentiana scabra Bunge.	Li et al. (2016)
148	Chrysoeriol	Gentiana scabra Bunge.	Li et al. (2016)
149	Isoorientin-7-O-glucoside	Gentiana scabra Bunge.	Deng (2015)
150	Luteolin-7-O-glucoside	Gentiana scabra Bunge.	Deng (2015)

### 2.3 Lignans

**FIGURE 6:**
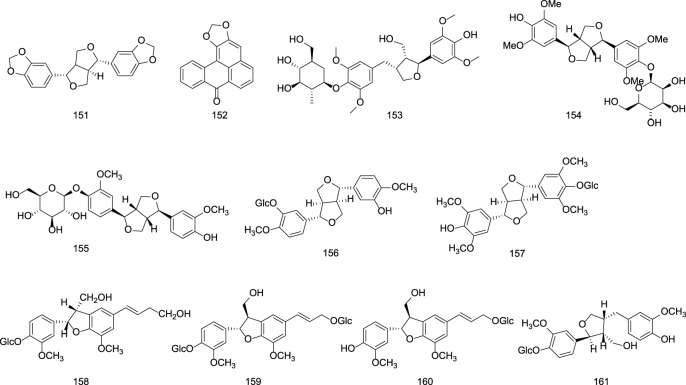
Chemical structures of Lignans in Gentianae Radix et Rhizoma.

Representative lignans isolated from *G. rigescens* include L-sesamin **(151)**, liriodendrin **(152)**, tortoside B **(153)**, and lignan glycosides such as (−)-syringaresinol-O-β-D-glucoside **(154)**, (−)-pinoresinol-O-β-D-glucoside **(155)**, syringaresinol-4′-O-β-D-glucopyranoside **(157)**, and lariciresinol-4-O-β-D-glucopyranoside **(161)** (Liu, 2004; Zhang et al., 2019; Ye et al., 2018). Specific information on lignans constituents from Gentianae Radix et Rhizoma can be found in Table 6 and Figure 6.

**TABLE 6:** Lignans in Gentianae Radix et Rhizoma.

No.	Compounds	Sources	Ref.
151	L-sesamin	*Gentiana scabra* Bunge.	Li (2023)
152	Liriodendrin	*Gentiana scabra* Bunge.	Li (2023)
153	Tortoside B	*Gentiana scabra* Bunge.	Li X. et al. (2018)
154	(-)-Syringaresinol-O-β-D-glucoside	*Gentiana yunnanensis*	Yang (2014)
155	(-)-Pinoresinol-O-β-D-glucoside	*Gentiana yunnanensis*	Yang (2014)
156	4,4′-Dimethoxy-3′-hydroxy-7,9′:7′,9-diepoxylignan-3-O-β-D-glucopyranoside	*Gentiana yunnanensis*	Yang (2014)
157	Syringaresinol-4′-O-β-D-glucopyranoside	*Gentiana yunnanensis*	Yang (2014)
158	Dehydrodiconiferyl alcohol-4-O-β-D-glucopyranoside	*Gentiana yunnanensis*	Yang (2014)
159	(7S,8R)-Balanophonin-4-O-β-D-glucopyranoside	*Gentiana yunnanensis*	Yang (2014)
160	(7S,8R)-Dehydrodiconiferyl alcohol-9′-O-β-D-glucopyranoside	*Gentiana yunnanensis*	Yang (2014)
161	Lariciresinol-4-O-β-D-glucopyranoside	*Gentiana yunnanensis*	Yang (2014)

### 2.4 Alkaloids

**FIGURE 7:**
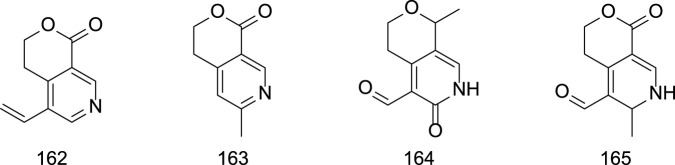
Chemical structures of Alkaloids in Gentianae Radix et Rhizoma.

Since the 1950s, several alkaloids, including gentianine **(162)**, gentialutine **(163)**, gentiamine **(164)**, and gentioflavine **(165)**, have been isolated from *G. rigescens* (Sun and Xia, 1984). However, studies suggest that these alkaloids may not be naturally occurring in the plant but rather artifacts formed during extraction. For instance, gentianine and gentialutine are proposed to arise from the conversion of gentiopicroside in the presence of ammonia during processing. Such interconversions between constituents not only influence the diversity and abundance of alkaloids but also modulate their pharmacological profiles. For example, gentianine exhibits significant anti-inflammatory, sedative, and antibacterial activities, whereas its precursor gentiopicroside primarily demonstrates hepatoprotective, anti-inflammatory, and immunomodulatory effects (Ning et al., 2017). Specific information on alkaloids constituents from Gentianae Radix et Rhizoma can be found in Table 7 and Figure 7.

**TABLE 7:** Alkaloids in Gentiana scabra Bunge.

No.	Compounds	Sources	Ref.
162	Gentianine	*Gentiana scabra* Bunge.	Ning et al. (2017)
163	Gentialutine	*Gentiana scabra* Bunge.	Ning et al. (2017)
164	Gentiamine	*Gentiana scabra* Bunge.	Ning et al. (2017)
165	Gentioflavine	*Gentiana scabra* Bunge.	Ning et al. (2017)

### 2.5 Other constituents

**FIGURE 8:**
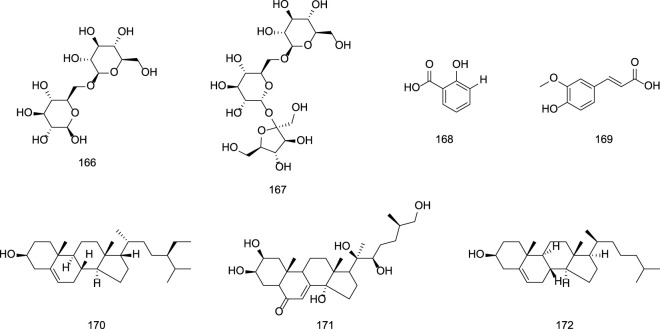
Chemical structures of other constituents in Gentianae Radix et Rhizoma.

Beyond terpenoids, flavonoids, lignans, and alkaloids, Gentianae Radix et Rhizoma contain diverse secondary metabolites. Polysaccharides such as gentiobiose **(166)** have been isolated (Wang, 2014; Jiang et al., 2008). Phenolic acids, including salicylic acid **(168)** and ferulic acid **(169)**, are also documented (Liu, 2004). Additionally, Gentiana contains amino acids (e.g., threonine, valine, methionine) and essential minerals such as calcium (Ca), copper (Cu), iron (Fe), zinc (Zn), and manganese (Mn) (Ye et al., 2023). Steroidal compounds, including β-sitosterol **(170)** and inokosterone **(171)**, have been identified (Zhao et al., 2009; Guo and Piao, 2011). The specific information is presented in Table 8 and Figure 8.

**TABLE 8:** Other constituents in Gentianae Radix et Rhizoma.

No.	Compounds	Sources	Ref.
166	Gentiobiose	*Gentiana scabra* Bunge.	Ye et al. (2023)
167	Gentianose	*Gentiana scabra* Bunge.	Ye et al. (2023)
168	Salicylic acid	*Gentiana scabra* Bunge.	Ye et al. (2023)
169	Ferulic acid	*Gentiana scabra* Bunge.	Ye et al. (2023)
170	Β-Sitosterol	*Gentiana scabra* Bunge.	Ye et al. (2023)
171	Inokosterone	*Gentiana scabra* Bunge.	Ye et al. (2023)
172	Cholesterol	*Gentiana scabra* Bunge.	Ye et al. (2023)

The identified iridoids and flavonoids not only contribute to the pharmacological efficacy but also serve as pivotal quality markers (Q-markers) for the standardization of Gentianae Radix et Rhizoma. Extraction methodologies across cited studies predominantly utilized methanol or ethanol (60%–95%) via reflux/maceration. For instance: Iridoids (e.g., gentiopicroside): 70% ethanol reflux, 2 h; Triterpenoids: Methanol maceration (48 h) silica gel chromatography; Polysaccharides: Hot water extraction (90 °C, 3 h); Comparative data suggest methanol outperforms ethyl acetate for iridoid yield (Δ = 12–18%), while ultrasound-assisted extraction reduces processing time by 40%.

Key compounds such as gentiopicroside, swertiamarin, and luteolin are critical for species authentication and ensuring batch-to-batch consistency, with HPLC-UV analysis typically requiring a relative standard deviation (RSD) of less than 5% for these benchmarks (Hu and Li, 2002). The establishment of these Q-markers bridges the chemical profiles with the efficacy, laying a foundation for quality control in pharmaceutical applications.

## 3 Biological activities

Gentianae Radix et Rhizoma, a traditional Chinese medicinal herb commonly known as Longdan, has been utilized for millennia to clear heat, dry dampness, and purge liver-gallbladder fire. Its therapeutic applications were first documented in the Shennong Bencao Jing (ca. 200 CE), where it was classified as a middle-grade herb with bitter-cold properties, noted for treating bone-interstice disorders, convulsions, and parasitic toxins, while enhancing cognition and longevity with prolonged use (Liu, 2022). Subsequent dynastic texts expanded its pharmacological profile. The Mingyi Bielu (Liang Dynasty) highlighted its efficacy in resolving gastric heat, seasonal febrile diseases, and heat-type diarrhea (Li et al., 2011), establishing its foundational role in heat-clearing and damp-drying therapies.

During the Song-Yuan period, the Taiping Shenghui Fang introduced the prototype of Longdan Xiegan Tang (Gentian Liver-Draining Decoction), combining gentian with Bupleurum and Scutellaria to address liver-gallbladder fire excess syndromes (Wan, 2022). Kou Zongshi’s Bencao Yanyi emphasized its morphological distinction as a short-rooted herb with intense bitterness (Zhu et al., 2021). By the Ming-Qing era, Li Shizhen’s Bencao Gangmu systematized its uses, including throat pain, wind-heat night sweats, and ocular inflammation, while noting enhanced efficacy through wine-processing (Li and Wang, 2023). The Yaopin Huiyi further refined its meridian tropism, specifying its action on liver-gallbladder fire and associated disorders such as ocular pain and pediatric convulsions (Zhang, 2015).

Modern pharmacological studies validate its bioactive potential, demonstrating anti-inflammatory, analgesic, hepatoprotective, choleretic, antitumor, antioxidant, and digestive properties (Liu et al., 2024; Li et al., 2024; Zhang et al., 2023; Li, 2023; Wang et al., 2023). The 2020 Chinese Pharmacopoeia lists six gentian-containing formulas, including Longdan Xiegan Wan and Qingre Jiedu Koufuye, reflecting its clinical versatility. Clinically, it is widely employed in managing herpes zoster, hypertension, sudden deafness, and inflammatory conditions, with Longdan Xiegan Tang remaining a cornerstone therapy for damp-heat liver disorders. Globally, gentian is recognized as a bitter stomachic, underscoring its cross-cultural pharmacological relevance (Yang and Wang, 2005).

This integration of historical wisdom and contemporary science positions Gentianae Radix et Rhizoma as a multifaceted medicinal agent, bridging traditional applications with evidence-based therapeutic potential.

### 3.1 Anti-inflammatory and analgesic effects

As a pivotal herb in traditional heat-clearing and damp-drying therapies, Gentianae Radix et Rhizoma has garnered significant attention for its anti-inflammatory and analgesic properties. Modern studies reveal that these activities stem from multi-target and multi-pathway synergistic mechanisms, involving inflammation mediator regulation, signaling pathway modulation, and neurotransmitter adjustment, with demonstrated efficacy across diverse disease models.

#### 3.1.1 Molecular mechanisms of anti-inflammatory action and disease intervention

The anti-inflammatory effects of Gentianae Radix et Rhizoma primarily target the NF-κB (Nuclear Factor κB) signaling axis. Experimental evidence confirms that its active component, gentiopicroside, inhibits IκB kinase β (IKKβ) phosphorylation, blocks NF-κB nuclear translocation, and subsequently downregulates key inflammatory mediators such as COX-2 (Cyclooxygenase-2) and TNF-α (Tumor Necrosis Factor-α). Gentiopicroside directly inhibits the phosphorylation of IKKβ, which prevents the degradation of IκB and subsequent nuclear translocation of the NF-κB p65 subunit. This blockade leads to the downregulation of key inflammatory mediators, including COX-2, TNF-α, and IL-6. Concurrently, gentiopicroside suppresses the activation of JNK and p38 within the MAPK pathway, further reducing the production of pro-inflammatory cytokines. In rheumatoid arthritis models, this mechanism significantly alleviates synovial inflammation and joint destruction. Concurrently, it suppresses JNK (c-Jun N-terminal Kinase)/p38 activation in the MAPK (Mitogen-Activated Protein Kinase) pathway, reducing macrophage secretion of IL-6 (Interleukin-6) and IL-1β (Interleukin-1β), thereby mitigating inflammatory infiltration in alcoholic liver injury. An *in vivo* study demonstrated that the ethyl acetate crude extract of Gentiana striata Maxim alleviated paw edema in a rat model of rheumatoid arthritis after 28 days of oral administration at doses of 100 and 200 mg/kg. This protective effect was associated with decreased levels of PGE_2_ and NO and was superior to that achieved with 100 mg/kg prednisone, a common anti-inflammatory drug (Cao et al., 2012). Notably, Gentianae Radix et Rhizomacomponents also remodel the inflammatory microenvironment via epigenetic regulation: in ulcerative colitis models, they promote macrophage polarization toward the M2 anti-inflammatory phenotype and inhibit histone deacetylase activity, enhancing chromatin accessibility of anti-inflammatory genes (Xie et al., 2021).

#### 3.1.2 Multi-dimensional regulation of analgesic effects

The analgesic mechanisms of Gentianae Radix et Rhizoma extend beyond conventional anti-inflammatory frameworks, exerting analgesic effects through a central-peripheral synergistic network. Centrally, gentiopicroside downregulates NR2B (N-Methyl-D-aspartate receptor subunit 2B) subunit expression of NMDAR (N-Methyl-D-Aspartate Receptor) in the anterior cingulate cortex, inhibiting glutamatergic synaptic transmission and blocking pain sensitization (Chen et al., 2008). Peripherally, it reduces substance P release in the spinal dorsal horn and activates the μ-opioid receptor pathway to stimulate endogenous analgesic substances such as β-endorphin (Jia et al., 2012). In neuropathic pain models, this multi-target approach significantly alleviates mechanical allodynia and thermal hyperalgesia without inducing tolerance typically associated with traditional opioids (Xie et al., 2021). Particularly in chronic inflammatory pain, Gentianae Radix et Rhizoma synergistically enhance anti-inflammatory and analgesic outcomes by inhibiting PGE2 (Prostaglandin E2) synthesis and TRPV1 (Transient Receptor Potential Vanilloid 1) channel activation, offering novel strategies for managing inflammatory bowel disease. The specific compounds with anti-inflammatory and analgesic effects, as well as their mechanisms of action, are presented in Table 9.

**TABLE 9:** Anti-inflammatory and analgesic compounds in Gentianae Radix et Rhizoma: Bioactive components and mechanisms of action.

No.	Active component	Chemical class	Mechanisms of action
6	Gentiopicroside	Iridoid	Inhibits IKKβ phosphorylation
			Blocks NF-κB nuclear translocation
			Downregulates COX-2, TNF-α
			Central: downregulates NMDAR-NR2B expression
			Peripheral: inhibits substance P release
			Peripheral: activates μ-opioid receptors
7	Loganin	Iridoid	Modulates inflammatory microenvironment
51	Swertiamarin	Iridoid	Suppresses JNK/p38-MAPK pathway, reduces IL-6 and IL-1β secretion
- ^1^	Gentianae Radix et Rhizoma	-	Inhibiting PGE2 synthesis and TRPV1 channel activation

^1^Not corresponding to any numbering in the preceding text.

### 3.2 Hepatoprotective and choleretic activities

Gentianae Radix et Rhizomaexerts hepatoprotective effects through a multidimensional “antioxidant-anti-inflammatory-metabolic modulation-anti-fibrotic” mechanism, while its choleretic activity is closely associated with bile acid transport regulation. Core constituents such as gentiopicroside and swertiamarin have emerged as pivotal lead compounds for liver disease drug development, particularly in alcohol-associated liver disease (ALD), metabolic dysfunction-associated steatotic liver disease, and hepatic fibrosis.

#### 3.2.1 Hepatoprotective mechanisms

Crude Gentianae Radix et Rhizomaextracts and gentiopicroside significantly reduce ALT (Alanine Aminotransferase) and AST (Aspartate Aminotransferase) levels in acute liver injury models induced by CCl_4_ or D-galactosamine, while enhancing glutathione peroxidase and superoxide dismutase (SOD) activity, reducing malondialdehyde (MDA) accumulation, directly scavenging free radicals, and reinforcing hepatic antioxidant defenses (Kikuchi et al., 2005). Swertiamarin accelerates toxicant metabolism by activating cytochrome P450 family 3 subfamily A member 4 (CYP3A4) and cytochrome P450 family 2 subfamily E member 1 (CYP2E1) enzymes, thereby attenuating CCl_4_-induced hepatotoxicity. Oral swertiamarin (100–200 mg/kg, 8 weeks) alleviated CCl_4_-induced hepatotoxicity in rats by activating the Nrf2/HO-1 pathway, reducing oxidative stress and inflammation (Wu et al., 2017). Additionally, Gentianae Radix et Rhizomacomponents suppress NF-κB signaling, downregulating hepatic pro-inflammatory cytokines such as TNF-α and IL-6, thereby ameliorating lipopolysaccharide-and *Bacillus* Calmette-Guérin-induced liver injury. In ALD models, gentiopicroside targets the P2X purinoceptor 7 (P2X7) receptor/NOD-like receptor thermal protein domain-associated protein 3 (NLRP3) inflammasome axis, inhibiting inflammasome activation, reducing lipogenesis, and promoting lipid oxidation to alleviate alcoholic steatosis (Li X. et al., 2018). Swertiamarin exerts its hepatoprotective effects by activating the Nrf2 antioxidant pathway. Furthermore, gentiopicroside targets the P2X7 receptor, inhibiting NLRP3 inflammasome assembly and subsequent IL-1β maturation.

#### 3.2.2 Anti-fibrotic interventions

Angiotensin system modulation: Swertiamarin inhibits AngII-AT1R signaling, blocking ERK (Extracellular Signal-Regulated Kinase)/c-Jun phosphorylation, thereby attenuating N-nitrosodimethylamine-induced hepatic stellate cell activation and fibrosis. The *in vitro* study using primary rat hepatic stellate cells showed that purified tetramethylpyrazine (5–20 μM, 12–24 h treatment) inhibited Ang II-induced activation, with DMSO as vehicle control and imatinib/rapamycin as positive controls (Zhang et al., 2014). This *in vitro* and *in vivo* study demonstrated that purified swertiamarin (Swe, 98% purity) at doses of 2.4–15 µM (in primary rat HSCs) and 15–20 mg/kg (in DMN-induced fibrotic rats) inhibited angiotensin II–induced activation and fibrosis, with losartan as a positive control and vehicle as negative control; treatment durations were 24 h (*in vitro*) and 2 weeks (*in vivo*), but no IC_50_/EC_50_ values were provided (Li et al., 2016). TGF-β1/Smad pathway regulation: G. rigescens suppresses TGF-β1/Smad2/3 signaling, downregulates connective tissue growth factor expression, reduces collagen deposition, and mitigates bleomycin-induced hepatic fibrosis (Li X. et al., 2018). Lipid metabolic balance: Gentiopicroside reduces triglyceride accumulation in ALD by modulating fatty acid synthase (FASN) and carnitine palmitoyltransferase 1 (CPT1) expression, restoring lipid oxidation-synthesis equilibrium. The study used acute and chronic alcoholic hepatosteatosis mouse models (40–80 mg/kg GPS) and ethanol-exposed HepG2 cells to show that purified genitopicroside (>99%) improved lipid metabolism via LKB1/AMPK activation and P2x7R–NLRP3 inflammasome inhibition. Metformin and A438079 served as positive controls, with treatments lasting 24 h (*in vitro*) to 10 days (*in vivo*); IC_50_/EC_50_ values were not provided (Lian et al., 2018).

#### 3.2.3 Choleretic mechanisms

Swertiamarin enhances bile acid efflux by upregulating bile salt export pump (BSEP) and multidrug resistance-associated protein (MRP) expression, improving cholestasis. Crude Gentianae Radix et Rhizoma extracts increase hepatocyte membrane fluidity, enhance hepatic microcirculation, and alleviate biliary dysfunction in thioacetamide-induced models. In cecal ligation and puncture (CLP)-induced septic liver injury, gentiopicroside protects hepatocyte mitochondrial function by inhibiting inducible nitric oxide synthase activity and reducing excessive nitric oxide production (Zhang et al., 2022). The hepatoprotective and choleretic compounds in Gentianae Radix et Rhizoma, along with their bioactive components and mechanisms of action, are displayed in Table 10.

**TABLE 10:** Hepatoprotective and Choleretic compounds in Gentianae Radix et Rhizoma: Bioactive components and mechanisms of action.

No.	Active component	Chemical class	Mechanisms of action
6	Gentiopicroside	Iridoid	Inhibits P2X7/NLRP3 inflammasome axis
			Modulates FASN/CPT1 lipid metabolism
51	Swertiamarin	Iridoid	Upregulates BSEP/MRP for bile acid efflux
			Activates CYP3A4/2E1 for detoxification
- ^1^	Gentianae Radix et Rhizoma	-	Increase hepatocyte membrane fluidity
			Enhance hepatic microcirculation
			Alleviate biliary dysfunction in thioacetamide-induced models

^1^Not corresponding to any numbering in the preceding text.

### 3.3 Antitumor activity

Gentianae Radix et Rhizomaexhibits broad-spectrum antitumor activity through multi-component interactions (e.g., gentiopicroside, polysaccharides, and Luteolin), targeting cell cycle regulation, apoptosis induction, autophagy modulation, and critical signaling pathway inhibition.

#### 3.3.1 Inhibition of tumor cell proliferation and cell cycle arrest

Gentiopicroside activates the p38/MAPK signaling pathway, upregulates pro-apoptotic B-cell lymphoma-2-associated X protein (Bax), downregulates anti-apoptotic B-cell lymphoma-2 (Bcl-2), and suppresses proliferation in human ovarian cancer HO8910 cells. It induces S-phase and G2/M-phase arrest in hepatocellular carcinoma HepG2 cells while increasing the G0/G1-phase population, effectively inhibiting proliferation. This compound also significantly reduces viability in human liver cancer SMMC-7721 and lung cancer A549 cells. Gentianae Radix et Rhizoma polysaccharides enhance thymic and splenic indices in tumor-bearing mice post-chemotherapy, boosting immune responses. Additionally, compounds such as gentiakochianin and gentiacaulein induce apoptosis in glioma U251 cells by reducing mitochondrial membrane potential and promoting reactive oxygen species (ROS) generation. Luteolin inhibits non-small cell lung cancer (NSCLC) proliferation and induces autophagy via suppression of the PI3K/Akt/mTOR/p70S6K signaling axis. Furthermore, the study demonstrated through *in vitro* assays that certain triterpenoids isolated from Gentiana scabra exhibit potent inhibitory activity against human indoleamine 2,3-dioxygenase (IDO), with the most active compounds showing IC_50_ values below 10 μM (Li et al., 2015).

#### 3.3.2 Multi-pathway antitumor effects

Crude Gentianae Radix et Rhizomaextracts inhibit proliferation in diverse cancer cell lines *in vitro*, including lung A549, colon HCT-116, prostate PC-3, and breast MCF-7 cells. *In vivo*, these extracts significantly suppress growth of mouse sarcoma S180 solid tumors, with medium- and high-dose groups showing tumor inhibition rates exceeding 30%. Specific compounds, such as chirat-16-en-3-one and chiratenol, demonstrate antiproliferative effects against cervical cancer HeLa cells. Globuloside A, cornusoside A, cornolactone A, 6,9-epi-8-O-acetylshanziside methyl ester, and polar extracts (petroleum ether, ethyl acetate) from Gentiana manshurica exhibit potent inhibition of HepG2 cell viability (Isakovic et al., 2008). The antitumor components in Gentianae Radix et Rhizoma, including their bioactive components and mechanisms of action, are presented in Table 11.

**TABLE 11:** Antitumor component in Gentianae Radix et Rhizoma: Bioactive components and mechanisms of action.

No.	Active component	Chemical class	Mechanisms of action
25	Gentiopicroside	Iridoid	Activates p38/MAPK pathway
			Upregulates bax
			Downregulates bcl-2
			Induces cell cycle arrest (S-phase, G2/M-phase, G0/G1-phase)
- ^1^	Gentianae Radix et Rhizoma polysaccharides	Polysaccharides	Enhances thymic and splenic indices
			Boosts immune responses in tumor-bearing mice
132	Gentiakochianin	Flavonoid	Induces apoptosis in glioma U251 cells by reducing mitochondrial membrane potential and promoting ROS generation
138	Luteolin	Flavonoid	Inhibits NSCLC proliferation
			Induces autophagy via suppression of PI3K/Akt/mtor/p70s6k signaling axis
- ^1^	Crude Gentianae Radix et Rhizoma extracts	Extract mixture	Inhibits proliferation in various cancer cell lines (A549, HCT-116, PC-3, MCF-7)
			Suppresses tumor growth in mouse sarcoma S180
98	Chirat-16-en-3-one	Triterpenoids	Antiproliferative effects against cervical cancer hela cells
99	Chiratenol	Triterpenoids	Antiproliferative effects against cervical cancer hela cells
15	Globuloside A	Iridoid glycoside	Inhibits hepg2 cell viability
16	Cornusoside A	Iridoid glycoside	Inhibits hepg2 cell viability
17	Cornolactone A	Iridoid	Inhibits hepg2 cell viability
18	6,9-epi-8-O-acetylshanziside methyl ester	Iridoid glycoside	Inhibits hepg2 cell viability

^1^Not corresponding to any numbering in the preceding text.

### 3.4 Regulation of gastrointestinal function

Gentiopicroside, a key bioactive component of *G. scabra*, improves gastrointestinal function through enhancing gastric motility, protecting gastric mucosa, and regulating gastrointestinal hormones.

#### 3.4.1 Promotion of gastric motility

Gentiopicroside ameliorates stress-induced gastrointestinal dysmotility in rat models by upregulating motilin receptor expression and downregulating vasoactive intestinal peptide receptor 2 (VIPR2) levels, thereby enhancing gastric emptying and intestinal peristalsis (Ruan et al., 2015).

#### 3.4.2 Gastric mucosal protection

In ethanol-induced gastric mucosal injury models, gentiopicroside upregulates heat shock protein-70 (HSP70), restores epidermal growth factor (EGF) and vascular endothelial growth factor (VEGF) levels, and promotes mucosal repair (Yang et al., 2018). The gastrointestinal-regulating bioactive compounds in Gentianae Radix et Rhizoma and their mechanisms of action are presented in Table 12.

**TABLE 12:** Gastrointestinal-Regulating Bioactive compounds in Gentianae Radix et Rhizoma: mechanisms of action

No.	Active component	Chemical class	Mechanisms of action
25	Gentiopicroside	Iridoid glycoside	Promotion of Gastric Motility: Upregulates motilin receptor expression, downregulates VIPR2 levels, enhances gastric emptying and intestinal peristalsis.
			Gastric Mucosal Protection: Upregulates HSP70, restores EGF and VEGF levels, and promotes mucosal repair in ethanol-induced injury models.

### 3.5 Regulation of the nervous system

The neuroregulatory properties of Gentianae Radix et Rhizomaare primarily mediated by its core constituent, gentiopicroside, which exerts neuroprotective, anti-neurodegenerative, and psychotherapeutic effects through multi-target synergy involving oxidative stress modulation, neurotransmitter regulation, and signaling pathway intervention.

#### 3.5.1 Neuroprotective effects

Antioxidant Synergy and Cell Survival: Bellidifolin, gentisides A/B, amarogentin, and gentiopicroside enhance neuronal survival under oxidative stress (e.g., H_2_O_2_-induced PC12 cells) by activating insulin receptor downstream pathways, including PI3K/Akt and Ras/Raf/ERK. These compounds elevate superoxide dismutase (SOD, SOD2) activity, reduce ROS and MDA levels, and mitigate oxidative neuronal damage (Deng, 2015). Inokosterone further attenuates mitochondrial dysfunction-mediated apoptosis in oxidative stress models.

#### 3.5.2 Cognitive enhancement

*G. rigescens extracts* enhance cognitive function in memory-impaired models by inhibiting acetylcholinesterase activity, thereby preserving acetylcholine levels, and by modulating the insulin-like growth factor 1 receptor and ERK signaling pathway (Ma, 2012).

#### 3.5.3 Antidepressant and analgesic effects

Gentiopicroside alleviates pain-depression comorbidity in reserpine-induced models by downregulating glutamate NMDA receptor subunit 2B (GluN2B) subunit expression of NMDA receptors in the basolateral amygdala, reducing glutamatergic excitotoxicity. It also restores monoamine neurotransmitter balance (e.g., serotonin, dopamine), demonstrating antidepressant efficacy (Deng, 2015; Ma, 2012).

#### 3.5.4 Anti-parkinsonian effects

In 6-hydroxydopamine-induced Parkinson’s disease models, gentiopicroside ameliorates motor deficits and tremors by inhibiting dopaminergic neuron degeneration in the nigrostriatal pathway, suppressing α-synuclein aggregation, and attenuating neuroinflammation (Yang, 2014).

#### 3.5.5 Anti-addictive effects

Gentiopicroside reduces morphine-induced addictive behaviors by modulating opioid receptor signaling and dopamine reward pathways, reversing drug dependence-associated neuroplasticity (Ma, 2012).

#### 3.5.6 Central nervous system stimulation

Early studies indicate that gentianine enhances central nervous system excitability in mice, potentially through γ-aminobutyric acid (GABA) ergic system modulation (Sun and Xia, 1984). The nervous system-regulating bioactive compounds in Gentianae Radix et Rhizoma and their mechanisms of action are presented in Table 13.

**TABLE 13:** Nervous system-Regulating Bioactive compounds in Gentianae Radix et Rhizoma: mechanisms of action.

No.	Active component	Chemical class	Mechanisms of action
25	Gentiopicroside	Iridoid	Antioxidant (activates PI3K/Akt pathway)
			Anti-Parkinsonian (inhibits α-synuclein aggregation)
			Antidepressant (modulates monoamine neurotransmitters)
			Alleviates pain-depression comorbidity (downregulates glun2b)
			Reduces morphine-induced addictive behaviors (modulates opioid receptor signaling and dopamine reward pathways)
- ^1^	Bellidifolin	Xanthone	Enhances neuronal survival via insulin receptor pathways (PI3K/Akt, Ras/Raf/ERK)
			Elevates SOD activity
			Reduces ROS and MDA levels
171	Inokosterone	Phytoecdysteroid	Attenuates mitochondrial dysfunction-mediated apoptosis in oxidative stress models
- ^1^	Gentisides A/B	Iridoid glycosides	Enhance neuronal survival under oxidative stress (activates insulin receptor pathways)
			Elevates SOD activity
			Reduces ROS and MDA levels
- ^1^	Amarogentin	Iridoid	Enhances neuronal survival under oxidative stress (activates insulin receptor pathways)
			Elevates SOD activity
			Reduces ROS and MDA levels
162	Gentianine	Alkaloid	Enhances central nervous system excitability
			Potentially through GABA ergic system modulation

^1^Not corresponding to any numbering in the preceding text.

### 3.6 Antioxidant activity

Reports in the literature indicate that flavonoids and iridoids in Gentiana possess antioxidant activity. Li Peiyuan et al. (Li PY. et al., 2018) used the 1,1-diphenyl-2-picrylhydrazyl (DPPH) radical to measure the antioxidant capacity of Gentiana extracts and found that flavonoid compounds extracted with different solvents can effectively scavenge DPPH radicals, with antioxidant activity being directly proportional to the total flavonoid content. Additionally, GSP-IIb, GSP-IIa and iridoid compounds isolated from the rhizomes of Gentiana can scavenge DPPH radicals, exhibiting stable antioxidant activity (Suyama et al., 2013). Gentirigeoside B exerts antioxidant effects by inhibiting the mTOR/Sch9/Rim15/Msn signaling pathway and enhancing autophagy (Xiang et al., 2022). Furthermore, *in vitro* assays of 60% methanol extracts from six Caucasian Gentiana species (herbs and roots) demonstrated antioxidant (DPPH, superoxide scavenging, lipid peroxidation inhibition) and digestive enzyme inhibitory (α-amylase/α-glucosidase) activities, with positive controls including trolox, quercetin, caffeic acid, and acarbose; activity strongly correlated with phenolic content, though no specific IC_50_ values or dose ranges for crude extracts were provided (Olennikov et al., 2019). The antioxidant bioactive compounds in Gentianae Radix et Rhizoma and their mechanisms of action are presented in Table 14.

**TABLE 14:** Antioxidant component in Gentianae Radix et **Rhizoma**: Bioactive components and mechanisms of action.

No.	Active component	Chemical class	Mechanisms of action
78	Gentirigeoside B	Iridoids	Inhibit mtor/Sch9/Rim15/Msn signaling pathway,
			Enhance autophagy
- ^1^	Flavonoid extracts	Flavonoids	Scavenge DPPH radicals, antioxidant activity is directly proportional to total flavonoid content
- ^1^	GSP-IIa	Proteoglycans	Scavenge DPPH radicals, exhibit stable antioxidant activity
- ^1^	GSP-IIb	Proteoglycans	Scavenge DPPH radicals, exhibit stable antioxidant activity

^1^Not corresponding to any numbering in the preceding text.

### 3.7 Other effects

*In vivo* studies in normal, glucose-hyperglycemic, and streptozotocin-induced diabetic rats demonstrated that the ethyl acetate fraction of a methanol extract of Gentiana olivieri aerial parts, and its isolated active constituent isoorientin (doses: 7.5–30 mg/kg), significantly reduced blood glucose levels, with a minimal effective dose of 15 mg/kg; positive controls included tolbutamide, negative controls received vehicle, and treatment durations ranged from acute (four to six h) to subacute (15 days) administration (Sezik et al., 2005). Gentianae Radix et Rhizoma extracts and have demonstrated the ability to reduce the activity of hepatic enzymes such as alanine aminotransferase, aspartate aminotransferase, and alkaline phosphatase, as well as modulate the bile acid receptor G protein-coupled bile acid receptor 1 (TGR5)/β-arrestin2/NF-κB signaling pathway, thereby delaying the progression of diabetic nephropathy in mice fed a high-fat diet (Ghazanfar et al., 2017; Xi et al., 2020). Gentiopicroside can be utilized to improve skin conditions with impaired epidermal barriers (Wölfle et al., 2017); it also inhibits adipogenesis by modulating the 3T3-L1 pathway (Choi et al., 2019). Gentianae Radix et RhizomaBunge. Exhibits significant activity against adenovirus type 5 (C type), human rhinovirus type B (subtype 14), and respiratory syncytial virus; the underground parts of Gentianae Radix et Rhizoma possess diuretic effects (You, 2017). The other bioactivities of Gentianae Radix et Rhizoma are presented in Table 15.

**TABLE 15:** Other activities of Gentianae Radix et Rhizoma.

Activity type	Active component	Mechanisms of action
Antiviral	Crude extracts	Inhibits adenovirus, rhinovirus, respiratory syncytial virus
Diuretic	Underground extracts	Promotes water-salt metabolism
Skin barrier repair	Gentiopicroside	Improves epidermal barrier function
Anti-diabetic nephropathy	Crude extracts	Modulates TGR5/β-arrestin2/NF-κB pathway

Notwithstanding its wide spectrum of pharmacological activities, it is crucial to consider its safety profile; traditional wisdom cautions against its use in cases of spleen-stomach deficiency with cold symptoms due to its potent bitter and cold nature (Zhu et al., 2021), while modern toxicological assessments indicate a relatively low acute toxicity (LD_50_ > 5 g/kg in mice) and an absence of genotoxic concern as per OECD guidelines.

## 4 Clinical applications

As a classic herb in traditional Chinese medicine (TCM) for clearing heat, drying dampness, and purging liver-gallbladder fire, Gentianae Radix et Rhizoma has demonstrated enduring clinical value through centuries of practice. Modern research on its compound formulations and bioactive constituents has expanded its applications beyond traditional liver-gallbladder damp-heat syndromes to dermatological, cardiovascular, gynecological, otolaryngological, and systemic disorders, establishing a modern therapeutic framework integrating “disease-pattern differentiation, formula compatibility, and mechanistic clarity”.

### 4.1 Dermatological disorders: herpes zoster and postherpetic neuralgia

Herpes zoster, caused by reactivation of varicella-zoster virus (VZV), is attributed to liver fire hyperactivity and damp-heat toxin accumulation in TCM. *G. scabra*-based formulas exert antiviral, immunomodulatory, and analgesic effects (Sun and Sun, 2007; Liu et al., 2021).

#### 4.1.1 Acute phase treatment

Longdan Xiegan Tang (Gentian Liver-Draining Decoction) (Cao, 2022; Wu and Chen, 2022; Zhang and Zhang, 2018): Combining Gentianae Radix et Rhizoma (as the sovereign herb) with Scutellaria and Gardenia, this formula alleviates herpetic pain and accelerates crust formation.

#### 4.1.2 Inhibition of VZV replication and viral load reduction

Downregulation of serum TNF-α and IL-6, upregulation of IL-12 and CD3+/CD4+ T cells to enhance Th1 immune responses. Modulation of neuropeptides (e.g., substance P, β-endorphin) to reduce neurogenic inflammation.Longdan Jiedu Tang (Gentian Toxin-Resolving Decoction) (Sun and Sun, 2007): Enhanced with Isatis root and Clerodendrum, this formula shows superior efficacy in bacterial co-infections by reducing IgE levels, suppressing mast cell degranulation, and inhibiting histamine release to alleviate pruritus.

#### 4.1.3 Postherpetic neuralgia management

Longdan Shenmai Zhijing Tang (Gentian-Ginseng Spasm-Relieving Decoction) (Shao et al., 2022): Targets residual damp-heat and qi-yin deficiency by inhibiting spinal dorsal horn glial cell activation, reducing IL-1β/TNF-α-mediated neuro-sensitization, and upregulating neurotrophic factors for nerve repair. Bloodletting Acupuncture Combined Therapy: Adjunctive use with modified Longdan Xiegan Tang enhances CD8^+^ T cell activity and cytotoxic T lymphocyte responses to eliminate pathogens and restore microcirculation.

#### 4.1.4 Special localizations

Ramsay Hunt Syndrome: Longdan Dai Xie Xiao Zhen Tang (Gentian-Indigo-Scorpion Rash-Resolving Decoction) achieves an 89.3% efficacy rate by suppressing VZV reactivation in trigeminal ganglia and reducing vestibular nerve edema, improving otalgia and facial paralysis (Zhang, 2009).

### 4.2 Cardiovascular disorders: Hypertension and complications

Longdan Xiegan Tang exemplifies integrated TCM-Western therapy for liver fire-induced hypertension, targeting mechanisms such as: Blood Pressure Regulation: Suppression of angiotensin II/AT1 receptor signaling to counteract vasoconstriction and myocardial fibrosis. Activation of eNOS/NO pathway and inhibition of endothelin-1 to restore endothelial function. Metabolic Syndrome Management: Reduces total cholesterol, low-density lipoprotein, and blood pressure variability through HMG-CoA reductase inhibition, bile acid excretion, and modulation of the autonomic nervous system, particularly by enhancing vagal tone (Li, 2023; Li, 2014; Li, 1998; Yang et al., 2022; Zheng and RUAN, 2018; Zuo et al., 2020).

### 4.3 Hepatobiliary disorders: chronic hepatitis B and fatty liver

#### 4.3.1 Chronic hepatitis B

Fufang Xiongdan Yigan Jiaonang (Compound Bear Bile Hepatitis Capsule) (Hu and Li, 2002): Inhibits Hepatitis B virus DNA replication and promotes HBeAg seroconversion by blocking viral transcription and enhancing interferon-γ responses. Modified Longdan Xiegan Tang: Reduces liver stiffness and fibrosis via TGF-β1/Smad3 pathway inhibition and matrix metalloproteinase activation (Liu et al., 2017; Lyu and Lin, 2018; Wang et al., 2017).

#### 4.3.2 Non-alcoholic fatty liver disease

Gentiopicroside activates PPARα/CPT1 to enhance fatty acid β-oxidation and suppresses SREBP-1c-mediated lipogenesis. Combined with lifestyle intervention, Longdan Xiegan Tang reduces hepatic steatosis and serum free fatty acids (Ge et al., 2022).

### 4.4 Gynecological inflammation: Cervicitis, vaginitis, and HPV infection

Cervicitis with HPV: Longdan Xiegan Tang downregulates cervical IL-8, TNF-α, and HPV E6/E7 oncoprotein expression while enhancing secretory IgA and dendritic cell antigen presentation (Dai et al., 2023; Zhou, 2012; Zhou et al., 2013a; Zhou et al., 2013b; Jiang, 2018). Bacterial Vaginosis: Modified formulations disrupt biofilms, inhibit bacterial adhesins, and restore vaginal microbiota (lactobacilli increased by three to four log units; pH < 4.5) (Liu et al., 2017; Lyu and Lin, 2018; Wang et al., 2017).

### 4.5 Otolaryngological and ophthalmic disorders

Otitis Media: Longdan Xiegan Capsules with tympanocentesis reduce middle ear effusion and TGF-β1/β2 levels via TLR4/MyD88/NF-κB pathway inhibition (Su and Mou, 2022). Chronic Rhinosinusitis: Corrects Th17/Treg imbalance by suppressing IL-17A/IL-22 and improving Lund-Kennedy endoscopic scores (Li, 2020). Herpetic Keratitis: Longdan Mingmu Tang (Gentian Vision-Clearing Decoction) with ganciclovir inhibits HSV UL54 gene expression and enhances corneal repair (Huo and Guo, 2000; Qu, 2022). Acupuncture adjunct therapy modulates trigeminal ganglion microRNAs (miR-155, miR-146a) to reduce recurrence (Lu and Su, 2018).

### 4.6 Emerging applications

Diabetic Nephropathy: Gentiana extracts attenuate renal fibrosis via TGR5 receptor activation, inhibiting tubular epithelial-mesenchymal transition (EMT) and NF-κB-driven inflammation (Jiang et al., 2019). Chronic Kidney Disease: Diuretic effects of Gentiana roots match furosemide in sodium excretion but with reduced potassium loss, offering safer edema management (Huang, 2020).

**FIGURE 9:**
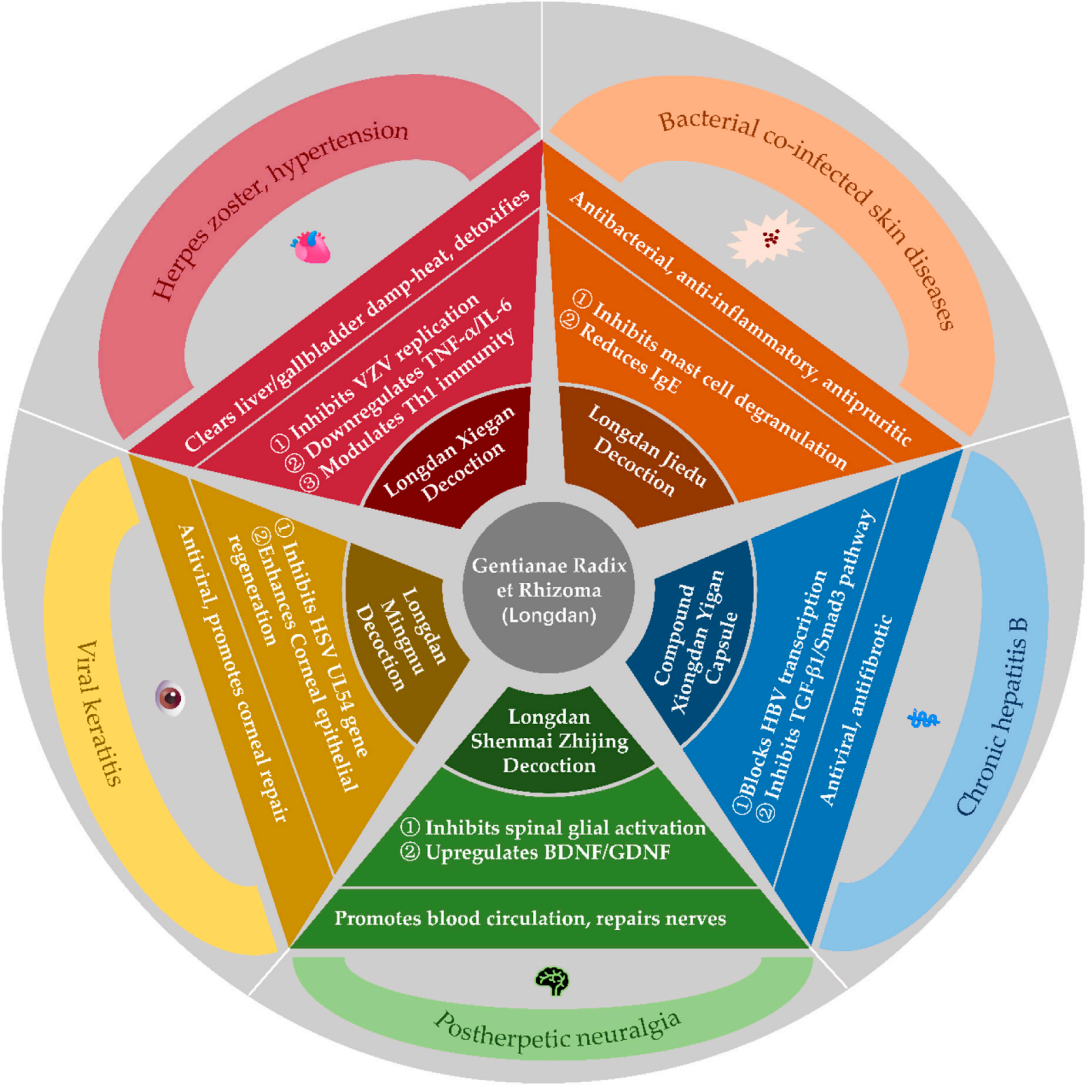
Clinical applications and Mechanisms of Gentianae Radix et Rhizoma and Its compound formulations.

The clinical applications and mechanisms of Gentianae Radix et Rhizoma and its compound formulations are illustrated in Figure 9.

## 5 Discussion and prospects

Gentianae Radix et Rhizoma, as a cornerstone of traditional and modern medicine, exhibits multifaceted therapeutic potential owing to its diverse chemical constituents and broad pharmacological activities. Despite significant advancements in identifying over 170 compounds, including terpenoids, flavonoids, and alkaloids, gaps remain in fully elucidating its chemical diversity. However, there is significant variation in efficacy between different species of Gentiana, such as *G. scabra* and *G. rigescens*. These species differ in their chemical profiles, which may affect their pharmacological actions. For instance, *G. scabra* is known for higher concentrations of certain iridoids like gentiopicroside, which contributes to its prominent anti-inflammatory and hepatoprotective properties. On the other hand, *G. rigescens* contains higher levels of specific triterpenoids, which may play a more substantial role in its antitumor and neuroprotective effects. These variations in chemical composition highlight the importance of considering species-specific differences when evaluating the therapeutic potential of Gentiana-based formulations. Advanced techniques such as metabolomics and AI-driven structural prediction could further uncover novel bioactive molecules, particularly minor or unstable constituents overlooked in conventional studies.

While the anti-inflammatory, hepatoprotective, and antitumor effects of key components like gentiopicroside and swertiamarin are well-documented, their molecular mechanisms—especially in modulating signaling pathways (e.g., TLR4/NF-κB, PI3K/Akt)—require deeper exploration. For instance, the interplay between gut microbiota and gentiopicroside’s neuroprotective effects or the epigenetic regulation of triterpenoids in fibrosis warrants systematic investigation.

Clinically, expanding applications beyond liver-gallbladder disorders to metabolic syndromes (e.g., diabetes, obesity) and neurodegenerative diseases (e.g., Parkinson’s, Alzheimer’s) represents a promising frontier. However, this necessitates rigorous clinical trials to validate efficacy and safety. Quality control remains a challenge; standardized protocols for active compound quantification, coupled with genomic and metabolomic fingerprinting, are critical to ensure batch consistency and minimize toxicity risks.

Furthermore, synergistic or antagonistic interactions between Gentiana extracts and conventional drugs (e.g., chemotherapeutics, antihypertensives) must be evaluated to optimize integrated therapies. Long-term toxicity studies and eco-friendly extraction methods should also be prioritized to align with sustainable pharmaceutical practices.

In summary, interdisciplinary collaboration—bridging phytochemistry, pharmacology, and clinical research—will unlock the full potential of Gentianae Radix et Rhizoma, transforming traditional wisdom into evidence-based solutions for global healthcare challenges.
